# Inhibiting homogeneous catalysis of cobalt ions towards stable battery cycling of LiCoO_2_ at 4.6 V†

**DOI:** 10.1039/d4sc07831d

**Published:** 2025-01-16

**Authors:** Chao Sun, Bing Zhao, Qing Wen, Xiang-tao Chen, Ning-yun Hong, Jin-bo Zeng, Jia-feng Zhang, Ling-jun Li, Jie-xi Wang, Xia-hui Zhang, Jun-chao Zheng

**Affiliations:** a School of Metallurgy and Environment, Central South University Changsha Hunan 410083 China jczheng@csu.edu.cn; b Engineering Research Center of the Ministry of Education for Advanced Battery Materials, Central South University Changsha 410083 China; c National Energy Metal Resources and New Materials Key Laboratory, Central South University Changsha 410083 China; d Key Laboratory of Comprehensive and Highly Efficient Utilization of Salt Lake Resources, Qinghai Institute of Salt Lakes, Chinese Academy of Sciences Xining 810008 China; e State Key Laboratory of Powder Metallurgy, College of Chemistry and Chemical Engineering, Central South University Changsha 410083 China; f School of Materials Science and Engineering, Changsha University of Science and Technology Changsha 410114 China; g National Engineering Research Centre of Advanced Energy Storage Materials Changsha 410205 China

## Abstract

Raising the cut-off voltage increases the energy density of LiCoO_2_ for lithium-ion batteries, but it exacerbates the decomposition of the electrolyte and the capacity decay of LiCoO_2_. To address such issues, many artificial cathode–electrolyte-interphases (CEIs) have been constructed to stabilize the cathode interface with an additive. However, electrolyte degradation by catalytic oxidation of Co ions dissolved in the electrolyte has rarely been explored. Herein, we report a new strategy of additive engineering towards enhanced cycling stability of LiCoO_2_ at 4.6 V. We found that the Co^4+^ ions dissolved in the electrolyte due to interfacial failure rapidly degrade the electrolyte by homogeneous catalysis, which can be deactivated by the chelation reaction of a nitrilotri(methylphosphonic acid) (ATMP) additive with Co^4+^. Benefiting from the deactivation of Co ions by ATMP, the catalytic oxidation of the electrolyte is suppressed, making the LiCoO_2_ interface more stable than the artificially constructed CEI, and thus the LiCoO_2_ cathode delivers a high capacity of 197.7 mA h g^−1^ after 200 cycles at 4.6 V with a retention rate of 91.4%. This work provides new insights into additive engineering towards stable cathode/electrolyte interfaces for next-generation batteries.

## Abstract 2

ATMP inhibits the homogeneous catalytic oxidation of Co ions by chelation coordination, preventing the decomposition of the electrolyte. Meanwhile, it can form a stable CEI film at the interface, slowing down the side reactions.

## Graphical abstract

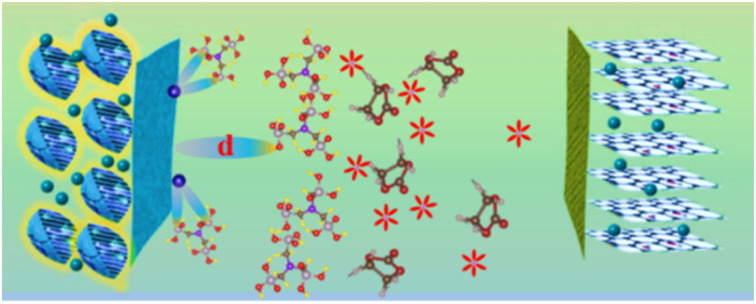

## Introduction

The deterioration in the climate and the environment in the 20th century accelerated the revolution in the energy field.^1^ With the rapid rise of artificial intelligence in the 21st century, the energy behind its large-scale computing power has further promoted the transformation of fossil energy into green and friendly energy, such as solar or wind. Meanwhile, this also places higher requirements on energy storage equipment supporting clean energy, especially lithium-ion batteries (LIBs), which are the main equipment for energy storage in electric vehicles and power stations.^2^ LiCoO_2_ (LCO), the most expensive component that makes up LIBs, is still widely used in high-end electronics due to its excellent tap density (4.2 g cm^−3^) and higher energy density (3.0–4.4 V, 2812 W h L^−1^) than LiFePO_4_ or LiNi_0.80_Co_0.10_Mn_0.10_O_2_.^3^ Furthermore, higher energy density can be achieved by increasing the charge cut-off voltage from 4.4 V to 4.6 V (220.0 mA h g^−1^, 3721 W h L^−1^),^4^ which can further break through the bottleneck of the device's demand for long endurance. However, when working under high-voltage conditions, a series of side reactions, such as decomposition of the electrolyte, generation of HF/LiF, and dissolution of cobalt, will become more severe than 4.4 V at the interface,^5^ which has a greater impact on capacity fading than an irreversible phase change (O3 to H1–H3 phase, 4.55 V).^6^ Thus, suppressing interfacial side reactions is key to improving the stability of LCO under high-voltage conditions.

In our previous studies,^7^ we confirmed the existence of heterogeneous catalytic behavior at the interface between LCO and the organic molecules of the electrolyte, and the catalytic reaction at the interface was alleviated by coating LCO with MXenes. On the other hand, a few studies have recently reported the oxidation of electrolyte by dissolved Co^4+^ ions caused by interface failure at high voltage, leading to a degradation in cycling performance.^8^ We recognize that Co complexes are widely used as homogeneous catalysts in the field of organic synthesis and degradation.^9^ Therefore, we infer that the Co ions dissolved in the electrolyte are likely to cause homogeneous catalytic degradation of the electrolyte, which has been overlooked and rarely explored in this field.

In this work, we explored the homogeneous catalytic behavior between Co^4+^ ions dissolved in the electrolyte and the electrolyte solvent ethylene carbonate (EC), which is regulated by electrolyte additive engineering. The results show that compared with traditional CEI additives, a more obvious improvement in the cycling stability of LCO can be produced by inhibiting the homogeneous catalytic behavior between Co^4+^ and electrolyte solvents with chelating additives. We found that the additive nitrilotri(methylphosphonic acid) (ATMP) has a high binding energy of −4.28 eV towards Co^4+^ ions, which effectively deactivates the homogeneous catalytic oxidation activity of Co^4+^ with EC by chelation coordination between ATMP and cobalt ions. As a result, when the battery is operated in electrolyte with ATMP additive, the LCO cathode delivers a specific capacity of 197.7 mA h g^−1^ at 4.6 V after 200 cycles, with a capacity retention rate of 91.4%. Moreover, the mitigation of electrolyte degradation by the ATMP additive is further verified by various characterization tools, such as *in situ* Raman, *in situ* electrochemical impedance spectroscopy (EIS), conductive atomic force microscopy (c-AFM), nuclear magnetic resonance (NMR), time of flight secondary ion mass spectrometry (TOF-SIMS), and soft X-ray absorption spectroscopy (sXAS).

## Results and discussion

**Fig. 1:**
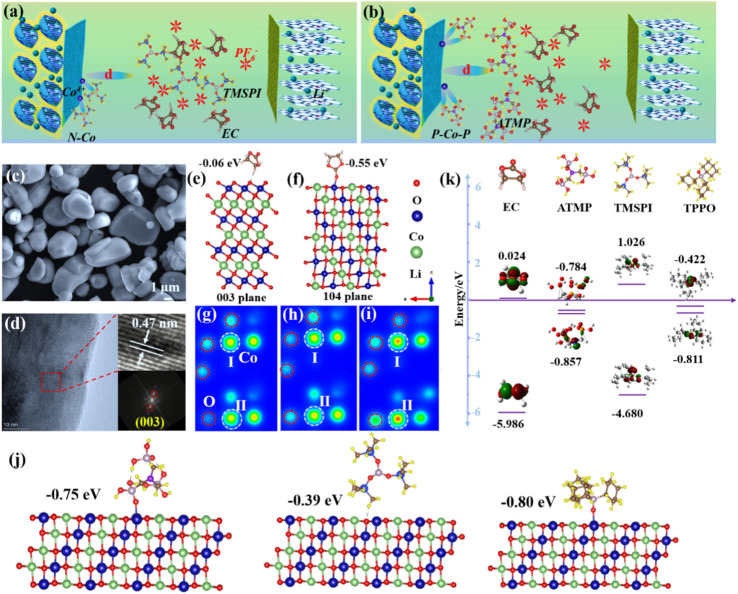
(a and b) Schematic decomposition model of electrolyte with LCO. (c and d) SEM and TEM images of LCO. (e and f) The calculated adsorption energy between the 003 plane/104 plane and EC. Charge density of (g) bare LCO, (h) the interaction model between the 003 plane and EC, and (i) the interaction model between the 104 plane and EC. (j) Adsorption energies and interaction sites between the three molecules and the 104 plane. (k) LUMO and HOMO of EC, ATMP, TMSPI, and TPPO.

Fig. 1a shows the working mode of a traditional film-forming agent. It is dispersed throughout the electrolyte system and preferentially oxidized at the interface to produce a stable CEI by relying on its highest occupied molecular orbital (HOMO) being higher than that of EC.^10^ However, this still cannot fundamentally alleviate the dissolution of cobalt ions during the charge and discharge process, triggering potential homogeneous catalytic behavior. In this study, the design of new additives should consider their unique functional groups to produce a strong bond between unique functional groups and cobalt ions, reduce the reaction energy of high-valent cobalt ions and prevent the electrolyte from being deeply oxidized (Fig. 1b). Fig. 1c shows that the size of the LCO particles is 6–10 μm, which is consistent with the result of particle size distribution in Fig. S1b.† XRD data is shown in Fig. S1a.† Meanwhile, the morphology and phase structure of the interface at the nanoscale are further illustrated by TEM in Fig. 1d. Lattice stripes with a spacing of 0.47 nm are observed at the edge of the particle, and the corresponding fast Fourier transform (FFT) patterns are also inserted in Fig. 1d, which demonstrate that LCO maintains a stable layered structure at the interface.^11^ This article uses three additive molecules as research objects. Tris(trimethylsilyl)phosphite (TMSPI) and triphenylphosphine oxide (TPPO) have been reported for use as artificial CEI additives to stabilize the interface of LCO. Nitrilotri(methylphosphonic acid) (ATMP) is a new additive designed by us. In order to shield the system from interference from heterogeneous catalysis between the cobalt-containing interface and the electrolyte, the active crystal plane for the reaction between lithium cobalt oxide and organic molecules was first determined.

Then, the binding energies between the three molecules and the active crystal face were also calculated. Density functional theory (DFT) was used to analyze the intensity of the interaction between the organic component of EC and the two main crystal planes of 003/104 (Fig. S1a†) to explore which crystal plane is dominant in the reaction. The reason for choosing EC is that EC is the most easily reactive molecule in the electrolyte component due to the low lower unoccupied molecular orbital (LUMO) of EC and its higher HOMO.^8^Fig. 1e shows that the mutual effect of EC and the 003 face is extremely weak: the adsorption value is only −0.06 eV, which indicates that there is almost no interaction between them. In contrast, the 104 plane reveals a stronger Co–O interaction (−0.55 eV) with EC than with 003 in Fig. 1f, which indicates that heterogeneous catalysis is more likely to occur on the 104 facet due to the large number of cobalt sites.^12^ Meanwhile, the Co–O bond also means that the activity of cobalt plays an important role in interfacial reactions, since the preferred binding site between organic molecules and LCO is the cobalt element. The exposure of Co ions enhances the reactivity of interfacial catalysis, as reported in our previous study.^7^ Moreover, this can be further illustrated by the charge density (Fig. 1g–i). Compared with bare LCO (Fig. 1g), the enhanced oxygen charge density signal also demonstrates that EC has weak interactions with the Co ions in LCO (Fig. 1h), and the enhanced cobalt signal shows that EC can produce strong bonding with cobalt on the 104 lattice (Fig. 1i). Therefore, the 104 facet was determined to be the active plane. After further calculation in Fig. 1j, the binding energies of the three molecules on the 104 facet are −0.75 eV (ATMP), −0.39 eV (TMSPI), and −0.80 eV (TPPO). The results demonstrate that the interaction force between ATMP and the interface is close to that of TPPO, and higher than that of TMSPI. In addition, their LUMO and HOMO are shown in Fig. 1k. The HOMO of ATMP (−0.857 eV) is higher than that of EC (−5.986 eV) or TMSPI (−4.68 eV), and close to that of TPPO (−0.811 eV). The electrolytes containing these four additives are noted as LED (bare), LAED (ATMP), LTMED (TMSPI), and LTPED (TPPO).

**Fig. 2:**
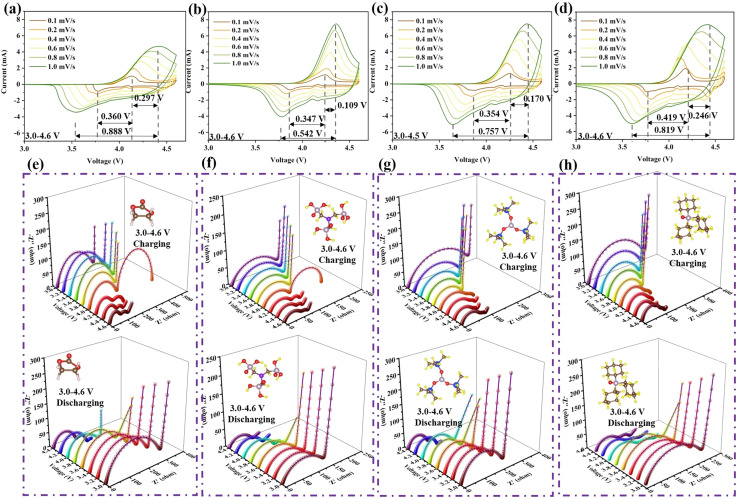
(a–d) Cyclic voltammetry curves at different scanning rates from 0.1 mV s^−1^ to 1.0 mV s^−1^ for the cells with (a) LED, (b) LAED, (c) LTMED, and (d) LTPED. *In situ* EIS testing during the charging and discharging process from 100 kHz to 10 Hz with different electrolytes: (e) LED, (f) LAED, (g) LTMED, and (h) LTPED under 3.0–4.6 V.

Cyclic voltammetry (CV) and *in situ* electrochemical impedance spectroscopy (EIS) were conducted to analyze the efficacy of the additives, as shown in Fig. 2. During the CV process, as the scanning speed increases, LED exhibits more severe polarization behavior than LAED, LTMED, or LTPED, which can be reflected in the distance between the oxidation and reduction peaks, where longer distance means greater polarization.^13^ LED shows the largest value of 0.888 V with a scanning rate of 1.0 mV s^−1^ in Fig. 2a. LAED displays the shortest distance of 0.542 V, which demonstrates that it can effectively alleviate the polarization phenomenon at the interface (Fig. 2b). Meanwhile, the smallest shift of the oxidation peak (0.109 V) further illustrates that LAED has the weakest polarization effect due to the stable CEI when the scanning rate increases from 0.1 mV s^−1^ to 1.0 mV s^−1^. However, compared with ATMP, TPPO exhibits a more severe degree of polarization as the scan speed increases. A larger distance of 0.246 V between the oxidation and reduction peaks was noticed at 1.0 mV s^−1^ (Fig. 2d). It is worth noting that since ATMP and TPPO have similar HOMO energy levels and interface forces, their properties should be consistent. However, the test results illustrate that the efficacy of the two is completely different. This means that the mechanism of interaction between them and LCO may be different.

In addition, interface impedance can also reflect the degree of electrolyte decomposition, because it can trigger the enrichment of insulating materials at the interface, resulting in an increase in the impedance value. Thus, *in situ* EIS was tested under 3.0–4.6 V, as shown in Fig. 2e–h. At the start of charging, the interface impedances (*R*_CEI_) of LED, LAED, LTMED, and LTPED are 273.5 Ω, 161.3 Ω, 252.4 Ω, and 313.3 Ω in Fig. S2a.† The performance of LTPED with the addition of agent TPPO is even worse than that of bare LED. As the voltage is increased to 4.6 V, the interface impedances of LED, LAED, LTMED, and LTPED decrease to 77.55 Ω, 50.34 Ω, 62.69 Ω, and 77.04 Ω, respectively. The LAED electrolyte enables LCO to maintain the minimum interface resistance during the entire charging process, as shown in Fig. 2f. The order of material interface resistance during the charging process for the four electrolyte systems is LAED < LTMED < LTPED < LED. As the voltage returns to 3.0 V, although the interfacial resistance of LCO under the four electrolyte conditions shows an increasing trend, LAED still maintains the minimum value. Their resistances are 288.3 Ω, 135.3 Ω, 217.9 Ω, and 227.3 Ω, respectively, in Fig. S2b.† The detection data of TMSPI also showed a discrepancy with the expected results. Compared with ATMP, the weak adsorption between TMSPI and the interface would reduce the heterogeneous catalytic reaction, so lower polarization and impedance should be observed. However, LTMED showed worse electrochemical performance. Therefore, further in-depth research is needed on the characteristics of the three molecules, especially their homogeneous catalytic behavior with cobalt ions dissolved in the electrolyte caused by interface failure. Additionally, it is noteworthy that the EIS charging profiles of LED and LAED above 4.0 V differ from those of LTMED and LTPED. The differences in the transport characteristics of ions and electrons may lead to significant inconsistencies in the EIS curve profile. Specifically, during the charging process, the initial reaction voltage plateau of LCO is 4.0 V. When the voltage reaches 4.0 V, LCO begins to participate in the electrochemical reaction, and the valence state of cobalt ions increases from +3 to +4, resulting in a significant enhancement of the oxidation properties of the cathodic interface. Therefore, the cause of this phenomenon may be that TMSPI and TPPO interfere with the transport pathway of solvated molecule EC-PF_6_^−^ in the electrolyte and the rate of insulating byproduct formation caused by its oxidation behavior at the interface.

**Fig. 3:**
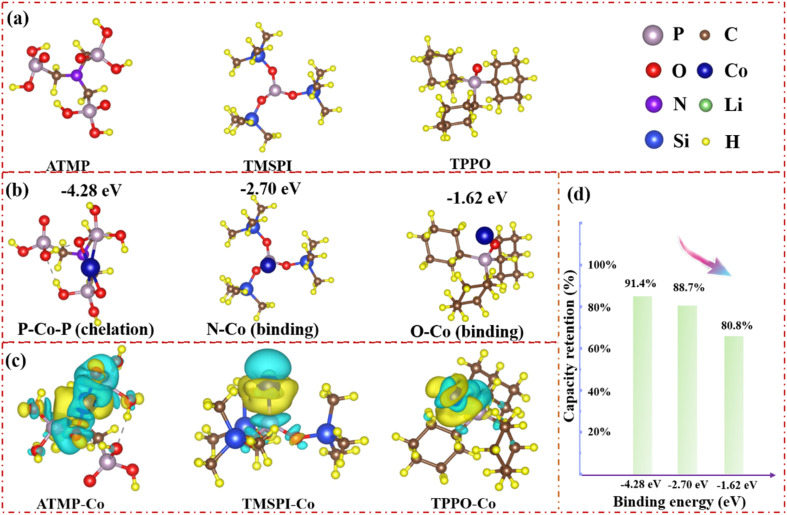
Density functional theory calculations related to ATMP, TMSPI, and TPPO. (a) The optimized structures of ATMP, TMSPI, and TPPO. (b) Calculated binding mode and structural energy between ATMP and Co ions, TMSPI and Co ions, and TPPO and Co ions. (c) Difference charge density map of ATMP–Co, TMSPI–Co, and TPPO–Co. (d) Relationship diagram between binding energy and capacity retention.

In addition to the intrinsic properties of the additives, in Fig. 3, DFT calculation illustrates the possible mechanism between Co ions and additives for the improvement in cycling stability. The structures of the three additive molecules were first optimized, as shown in Fig. 3a. The oxidation activity of cobalt ions may be an important factor in the homogeneous catalytic reaction. Therefore, exploring the inhibitory effect of additive molecules on the oxidation activity of cobalt ions can indirectly evaluate the degree of catalytic reaction. Fig. 3b shows that ATMP can reduce the oxidation of cobalt ions through the strong coordination effect of chelation, and the binding energy of −4.28 eV is the largest amongst the three additives, which means it was able to minimize the activity of the cobalt ions. TMSPI also shows strong binding ability to a high-valent Co^4+^ ion with a value of −2.70 eV, which means that TMSPI can also well resist the oxidation of Co^4+^. However, the binding energy between TPPO and Co is only −1.62 eV, which means that TPPO cannot effectively deactivate the activity of cobalt ions. This is an important reason why the polarization behavior and interface impedance of LTMED are stronger than those of LTPED. Moreover, the suppression of cobalt ion activity also improves the cycling stability of LCO in Fig. 3d. Data for the long-term cycling performance of a coin cell is shown in Fig. S3† with 3.0–4.6 V at 0.5C. LAED still reveals the highest capacity retention of 91.4%, with LTMED at 88.7%, LTPED at 80.8%, and LED at only 76.3%. This means that the homogeneous catalytic activity of cobalt ions can be significantly reduced through chelation coordination, thereby significantly inhibiting the decomposition of the electrolyte and greatly improving the cycle stability of the battery. The trend of capacity retention after 100 cycles of the full cell is consistent with that of the half cell at 4.5 V: 21.6% (LED), 83.5% (LAED), 77.2% (LTMED), and 59.8% (LTPED) (Fig. S4†). In addition, the differential charge density distribution of the three molecules combined with cobalt ions is shown in Fig. 3c. The yellow area represents the consumption of electrons, and the blue area represents the accumulation of electrons. ATMP–Co shows the maximum consumption of P electrons and accumulation of Co electrons due to the maximum overlap between the charge-increasing region and the charge-decreasing region, which indicates a maximized interaction between ATMP and Co^4+^ due to the chelation effect. Compared with TPPO–Co, TMSPI–Co displays a higher overlap area between the charge-increasing region and the charge-decreasing region, which also further demonstrates that with the strengthening of the Co–O bond (ATMP–Co > TMSPI–Co > TPPO–Co), the activity of cobalt ions gradually weakens, which can effectively alleviate the homogeneous catalysis between the electrolyte components.

**Fig. 4:**
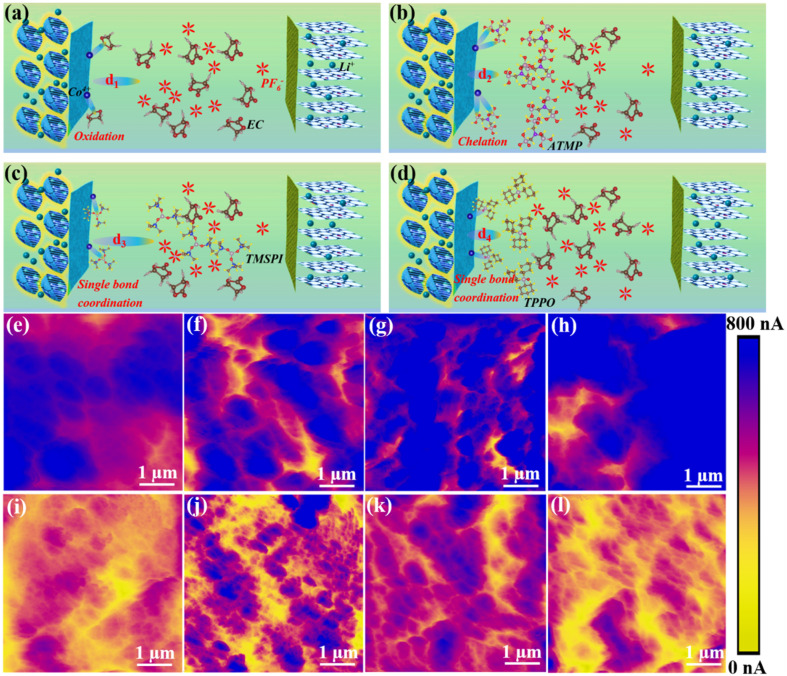
Interfacial interaction model. (a) EC and cathode, (b) ATMP and cathode, (c) TMSPI and cathode, (d) TPPO and cathode. *Ex situ* c-AFM detection of interface conductivity of cathode after 1 cycle with different electrolytes: (e) LED, (f) LAED, (g) LTMED, and (h) LTPED. The conductivity of the cathode interface after 200 cycles also detected with different electrolytes: (i) LED, (j) LAED, (k) LTMED, and (l) LTPED.

Fig. 4a–d illustrate the interaction mode between the four electrolytes and the interface during the charge and discharge process. Benefiting from the special functional groups of ATMP, the high HOMO value, and the strong binding energy with the 104 facet, ATMP can inhibit the catalytic oxidation activity of cobalt ions through chelation and can also preferentially oxidize at the interface to form a stable CEI, thereby improving the cycle stability of the system (Fig. 4b). The behavior of TPPO is similar to that of TMSPI since it has the same bonding model, but it cannot effectively inhibit the catalytic activity of cobalt ions (Fig. 4d). Compared with TPPO, the electrolyte of LTMED, having stronger binding energy with a Co ion (−2.70 eV), exhibits better performance due to the ability of TMSPI to effectively mitigate the activity of cobalt ions (Fig. 4c). Since EC-PF_6_^−^ has a higher HOMO value, which makes it easily oxidized, triggering further side reactions, the bare electrolyte exhibits the worst performance (Fig. 4a).^5*a*,14^ The high HOMO of ATMP (−0.857 eV), coupled with the strong adsorption energy (−0.75 eV), promotes the preferential adsorption and oxidation of ATMP at the LCO interface to form a stable CEI, which can prevent direct contact between EC-PF_6_^−^ and the interface with LCO, thereby avoiding over-oxidation of EC-PF_6_^−^.

The electrical conductivity of the interface can further serve as an indicator of the extent of electrolyte decomposition due to the decomposition of organic components and the formation of insulating material such as LiF, which can lead to a decrease in the conductivity of the interface, which is detected by AFM with a conductive-AFM model, as shown in Fig. 4e–l. Fig. 4e–h illustrate the distribution of the interface conductivity of LCO after one cycle in four electrolyte environments (LED, LAED, LTMED, LTPED). The corresponding current values are 400–800 nA, 550–800 nA, 500–800 nA, and 600–750 nA. Most of the blue areas in Fig. 4e to h show good electrical conductivity owing to the electrolyte not yet having undergone obvious decomposition in the initial stage of cycling. However, the blue area is obviously reduced after 200 cycles in Fig. 4i (200–300 nA) and Fig. 4l (200–450 nA), which can be attributed to the inability of LTPED and LED to effectively inhibit the occurrence of interfacial side reactions, especially for LED, resulting in an increase in the decomposition behavior of organic components and interface impedance. In contrast, Fig. 4j and k demonstrate excellent current strengths due to the blue area being clearly observable, where the ranges of current strength are 350–750 nA and 300–700 nA, respectively. This conclusion further illustrates that the decomposition of organic components and LiPF_6_ in LAED has been significantly inhibited.

**Fig. 5:**
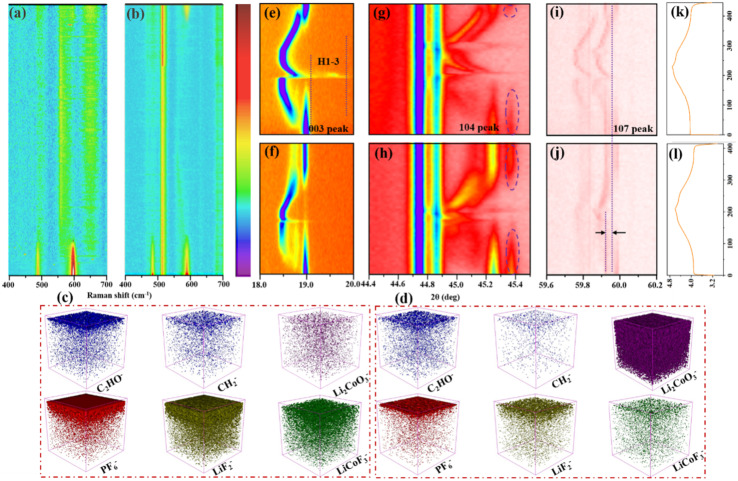
(a and b) *In situ* Raman measurement for cells containing (a) LED and (b) LAED under 3.0–4.6 V. (c and d) The content of molecular fragments of C_2_HO^−^, CH_2_^−^, LiCoO_3_^−^, PF_6_^−^, LiF_2_^−^, and LiCoF_3_^−^ at the interface of the cathode after 200 cycles from 3.0 V to 4.6 V with (c) LED and (d) LAED electrolyte, respectively. *In situ* XRD measurement for the cell containing LED with (e) 003 peak, (g) 104 peak, and (i) 107 peak under 3.0–4.6 V. *In situ* XRD measurement for the cell containing LAED with (f) 003 peak, (h) 104 peak, and (j) 107 peak at 3.0–4.6 V. (k and l) Corresponding charge–discharge curves.

To explore the effect of side reactions on interfacial attenuation and phase structure, *in situ* Raman, time of flight secondary ion mass spectrometry (TOF-SIMS), and *in situ* XRD were tested after 50 cycles, as shown in Fig. 5. The Raman and XRD raw data are displayed in Fig. S5 and S6.† At the beginning of charging, two characteristic peaks are observed at 485 cm^−1^ and 595 cm^−1^ in the Raman spectra (Fig. 5a–b), which can be attributed to O–Co–O (E_g_) and Co–O (A_1g_), respectively.^15^ These two peaks reflect the interface strength of Co–O during the charge and discharge process. The two peaks gradually disappeared during the charging process to 4.6 V in Fig. 5a and b. However, as the voltage is further discharged to 3.0 V, E_g_ and A_1g_ from the LCO interface of the LED system cannot be restored at all in Fig. 5a. Compared with LED, although the E_g_ signal was not observed in LCO from the LAED system, A_1g_ is clearly observed. This indicates that LCO combined with LAED can effectively eliminate side reactions at the interface and avoid corrosion of the cathode from outside to inside.^2*b*,16^ TOF-SIMS further confirms the results of *in situ* Raman spectroscopy. After 50 cycles, the contents of molecular fragments C_2_HO^−^, CH_2_^−^, LiCoO_3_^−^, PF_6_^−^, LiF_2_^−^, and LiCoF_3_^−^ on the interface of LCO, which was extracted from LED, were detected in Fig. 5c. C_2_HO^−^ and CH_2_^−^ represent the degree of decomposition of the electrolyte and the components of the CEI.^17^Fig. 5c shows thicker C_2_HO^−^ (3–4 nm) and CH_2_^−^ (2–3 nm) compared to Fig. 5d, which indicates that the decomposition of organic components in the electrolyte LED is more intense. Fig. 5d shows that the thickness of C_2_HO^−^ is only 2–3 nm and the thickness of CH_2_^−^ is 1 nm, due to ATMP alleviating the decomposition of the electrolyte by inhibiting interfacial oxidation behavior. Furthermore, the violent reaction at the interface will further accelerate the decomposition of LiPF_6_ because of PF_6_^−^ can overcome the electrostatic attraction with EC-Li^+^ around the anode and diffuse to the interface of the cathode, and combine with the highly polarized EC to form solvated ions of EC-PF_6_^−^, which can be easily oxidized.^8^ PF_6_^−^, LiF_2_^−^, and LiCoF_3_^−^ represent the decomposition of LiPF_6_ and corrosion of the material interface by HF. Fig. 5c shows that the thickness of PF_6_^−^, LiF_2_^−^, and LiCoF_3_^−^ is 3–5 nm, 4–6 nm, and 5–7 nm, respectively. Fig. 5d shows the thinner thickness of PF_6_^−^ (2–3 nm), LiF_2_^−^ (3–4 nm), and LiCoF_3_^−^ (2–3 nm) than in Fig. 5c. Meanwhile, Fig. 5d also reveals the stronger signal of LiCoO_3_^−^ than that of Fig. 5c, which symbolizes material integrity. Moreover, the content of organic components at the interface of the anode was captured by DMSO-d_6_ solvent to form a solution and analyzed by liquid nuclear magnetic resonance (NMR) in Fig. S7.† Compared with LAED, ^1^H NMR and ^19^F NMR display higher levels of H_2_O (3.4 ppm) and HF (−171.7 ppm) at the anode interface containing the LED system in Fig. S7b and S7d,† which also demonstrates that the decomposition of LAED electrolyte is significantly inhibited.

To explore the influence of interfacial reactions on the bulk structure, *in situ* XRD was further tested in Fig. 5e–j. The variation in the 003 peaks during the charge and discharge process is shown in Fig. 5e and f as an important basis for judging irreversible phase change at 0.5C. The shift of the 003 peak in LED (0.72°) is significantly larger than that in LAED (0.08°) when the voltage reaches 4.6 V, indicating that the phase transition from O3 to H1–3 in LED is more severe. In addition, it can clearly be seen that the 003 peak remains during the charge process, which can be attributed to the fact that the high charge/discharge current density (0.5C) causes less XRD data to be available during the *in situ* XRD test, which ultimately leads to hysteresis when the peaks are rendered. At a lower charge/discharge current density of 0.1C, the device collects more XRD data and the hysteresis is mitigated, which has been illustrated by the *in situ* XRD data of the 003 peak at 0.1C in Fig. S8.† Moreover, a peak was observed at 18.8° during the charging process, caused by splitting of the 003 peak and hysteresis of the peak caused by rendering.^18^ The changing trend of the 107 peaks is consistent with the conclusion about 003. The 107 peak of LAED shows almost no change during the circulation process in Fig. 5i, and the value of LED is about 0.04° (Fig. 5j). Meanwhile, compared with LED, the 104 peak of LAED appears earlier and the signal is stronger after one cycle, showing better structural recovery in Fig. 5h. These results illustrate that the stability of LAED is much higher than that of LED.

In addition, the XPS data of F-element after cycling is shown in Fig. S9,† where the results show that in the LAED electrolyte system, the F content at the cathode interface is lower than LED electrolyte after cycling, and the contents before and after sputtering are 26.27% and 25.92%, respectively, which indicates that the decomposition of LiFP_6_ is obviously suppressed. Meanwhile, the dissolution behavior of cobalt was further tested using ICP after soaking electrodes with DMC, which shows that the cobalt content in the CEI of the LAED electrolyte system is only 18 mg L^−1^, and that of the LED electrolyte system is 46 mg L^−1^. Values for both LTMED and LTPED are higher than that for LAED, but lower than that for LED, at 33 mg L^−1^ and 28 mg L^−1^, respectively (Fig. S10†). This can be further proved by O K-edge sXAS in Fig. S11,† where peaks “A” and “B” are attributed to Co^3+^ (e_g_)–O 2p and Co^4+^ (e_g_)–O 2p respectively.^19^ After 50 cycles, B is clearly present in LED, but not observed in LAED, which suggests that LAED is able to effectively protect the LCO interface by mitigating side reactions.

## Conclusions

In summary, this work reports that ATMP can effectively inhibit the catalytic oxidation activity of Co ions through chelating coordination, thereby mitigating the decomposition of the electrolyte. With the enhancement in the binding energy (Co–O) between the additive and cobalt ions, the catalytic oxidation activity of the cobalt ions gradually decreased. We found that the inhibitory effect of electrolyte degradation on the homogeneous catalytic activity of the cobalt ions is better than the addition of CEI film-forming agent in terms of improving the cycling stability of the LCO. Compared with the bare LED electrolyte, the electrolyte containing ATMP additive increased the capacity retention from 76.3% to 91.4% after 200 cycles. The LCO cycled in LAED has a thinner organic layer, lower impedance, and more stable structure at the interface after 200 cycles, which was verified by TOP-SIMS, c-AFM, *in situ* Raman, and *in situ* EIS. We anticipate that this work will provide a new strategy for effectively mitigating such electrolyte degradation as well as a significant reference for other cathode materials, especially for NCM, which has a similar structure and interfacial challenges to LCO, and thus will guide the rational design of new electrolytes for next-generation lithium-ion batteries with high energy density and long-term stability.

## Data availability

The data that support the findings of this study are available within the paper and its ESI Files.† Should any raw data files be needed in another format they are available from the corresponding author upon reasonable request.

## Author contributions

Chao Sun: writing – original draft, analyzing the research data, performing the experiment, and contributing significantly to the analysis and manuscript preparation. Bing Zhao: contributing to the physico–chemical analysis of as-prepared sample. Qing Wen: AFM characterization and the corresponding data analysis. Xiangtao Chen: devoting to the electrochemical result analysis. Ningyun Hong: contributing to the spectra analysis as mentioned in this paper. Jinbo Zeng: devoting to the designing of experiments and revising of the manuscript writing-English. Jiafeng Zhang: contributing to the electrochemical analysis of this paper. Lingjun Li: devoting to the graph processing. Jiexi Wang: contributing to the experiment assistance and English grammar check. Xiahui Zhang: contributing to the formation main structure of this research paper. Junchao Zheng: experiment designing, research data analysis and manuscript designing.

## Conflicts of interest

There are no conflicts to declare.

## Supplementary Material

SC-OLF-D4SC07831D-s001
