# Supplementary material for: Inhibiting homogeneous catalysis of cobalt ions towards stable battery cycling of LiCoO2 at 4.6 V
Source: Chem Sci. 2025 Jan 16;16(11):4842–50. doi: 10.1039/d4sc07831d (PMC11808795; doi:10.1039/d4sc07831d)
Supplement: SC-016-D4SC07831D-s001 [file SC-016-D4SC07831D-s001.pdf]

Supplementary Information (SI) for Chemical Science.
This journal is © The Royal Society of Chemistry 2025

Inhibiting Homogeneous Catalysis of Cobalt Ions towards Stable Battery Cycling of $LiCoO_2$ at 4.6 V

Chao Sun[1,2,3], Bing Zhao[4], Qing Wen[1,2,3], Xiang-tao Chen[1,2,3], Ning-yun Hong[5], Jin-bo Zeng[4], Jia-feng Zhang[1,2,3], Ling-jun Li[6], Jie-xi Wang,[1,2,3,7], Xia-hui Zhang[1,2,3], Jun-chao Zheng[1,2,3*]

[1].School of Metallurgy and Environment, Central South University, Changsha, Hunan 410083, China

[2].Engineering Research Center of the Ministry of Education for Advanced Battery Materials, Central South University, Changsha 410083, China

[3].National Energy Metal Resources and New Materials Key Laboratory, Central South University, Changsha 410083, China

[4].Key Laboratory of Comprehensive and Highly Efficient Utilization of Salt Lake Resources, Qinghai Institute of Salt Lakes, Chinese Academy of Sciences, Xining 810008, China

[5].State Key Laboratory of Powder Metallurgy, College of Chemistry and Chemical Engineering, Central South University, Changsha 410083, China.

Corresponding Authors (E-mail): jczheng@csu.edu.cn (j. c. Zheng)

Experimental Section

Sample Preparation of particle LCO, and different Types of Electrolytes: The target product, particle LCO, needs to be prepared by sintering Co_3O_4 with a particle size of 6-10 μm and Li_2CO_3 at a high temperature of 1000°C for 10 hours with a molar ratio of 1:1.55. LED electrolyte contains 1.0 M $LiPF_6$ in ethylene carbonate/dimethyl carbonate (volume ratio of EC:DMC = 1:1). LAED electrolyte contains 1.0 M $LiPF_6$ and 0.05 M nitrilotri(methylphosphonic acid) (ATMP) in ethylene carbonate/dimethyl carbonate (volume ratio of EC:DMC = 1:1). LTMED electrolyte contains 1.0 M $LiPF_6$ in tris(trimethylsilyl)phosphite/ethylene carbonate/dimethyl carbonate (volume ratio of TMSPI:EC:DMC = 0.5:4.0:5.0:0.5). LTPED electrolyte contains 1.0 M $LiPF_6$ and 0.05 M

triphenylphosphine oxide (TPPO) in ethylene carbonate/dimethyl carbonate (volume ratio of EC:DMC = 1:1).

Physical characterization: Scanning electron microscopy (JSM-7900F, JEOL, SEM) and High-resolution transmission electron microscopy (HRTEM, Thermo Fischer Talos F200x) were used to observe the morphology and interphase structure of large particle and small particle, respectively. The data of X-ray diffraction and In-situ X-ray diffraction were obtained using D8 Advance (XRD, Bruker) with Cu Kα (λ = 1.54 Å) radiation. The particle size was measured by Autosorb IQ2 (Quantachrome, US). Liquid nuclear magnetic resonance (LNMR, AVANCE III 500M) were also employed to show the decomposition degree of electrolyte. The attenuation degree of material interface was monitored using In-situ Raman spectra (Renishaw inVia, U.K.) (λ = 532 nm). The conductivity of the interface and the distribution of organic fragment molecules on the surface of cathode after 200 cycles were analyzed by Conductive-Atomic force microscope (c-AFM, Dimension ICON) and Time of flight secondary ion mass spectrometry (PHInanoTOF II), respectively.

Electrochemical characterization: The coin cell (CR2023) was tested to show the properties of Long-term cycling, Rate, Cyclic voltammetry (CV), and In-situ electrochemical impedance spectroscopy (In-situ EIS). Here, the cathode slurry consists of large particle/small particle (LCO), polyvinylidene difluoride (PVDF), and super p and comes with 8:1:1 mass ratio. After drying at a temperature of 60°C for 10 hours and rolling with pairs of rollers, the target electrode is finally produced. The polypropylene membrane is separator, anode is Li metal piece, electrolyte are LED, LAED, LTMED, and LTPED respectively. Long-term cycling was tested on the LAND test system (CT2001A, China). In-situ electrochemical impedance spectroscopy (In-situ EIS) from 100 kHz to 0.01 Hz, and Cyclic Voltammetry (CV) with a scan rate from 0.1 mV s^{-1} to 1.0 mV s^{-1} were collected on electrochemical workstation (Bio-Logic, VMP-300, France). For the full-cell test, the cathode is composed of 98% (LCO):1% (PVDF):0.5% (Carbon nanotubes, CNT):0.5% (super p); the anode is composed of 95% (graphite, ZET H9-5, China):2.0% (SBR binder):1.65% (super p):1.35% (CMC suspending agent); the membrane separator is PP, and the thickness is 0.012 mm. The cathodic press density was designed as 4.0 g cm^{-2}. And the anodic press density was designed as 1.5 g cm^{-2}. LED,

LAED, LTMED, and LTPED were used as electrolyte, respectively. The full-cells were tested at 0.5 C in a voltage range of 3.0-4.5 V.

Theoretical calculation: For the calculation parameters and procedures of density functional theory, please refer to our recently published article.[1]

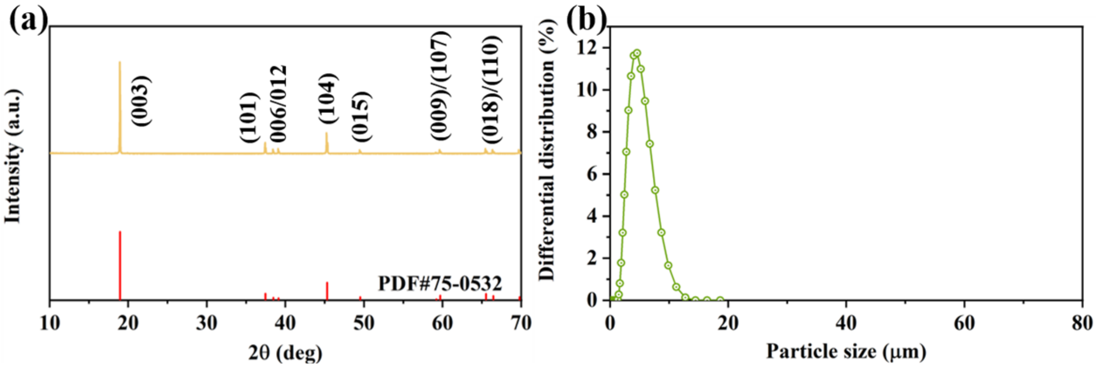

Figure S1. (a) XRD patterns of LCO, (b) Particle size distribution of LCO.

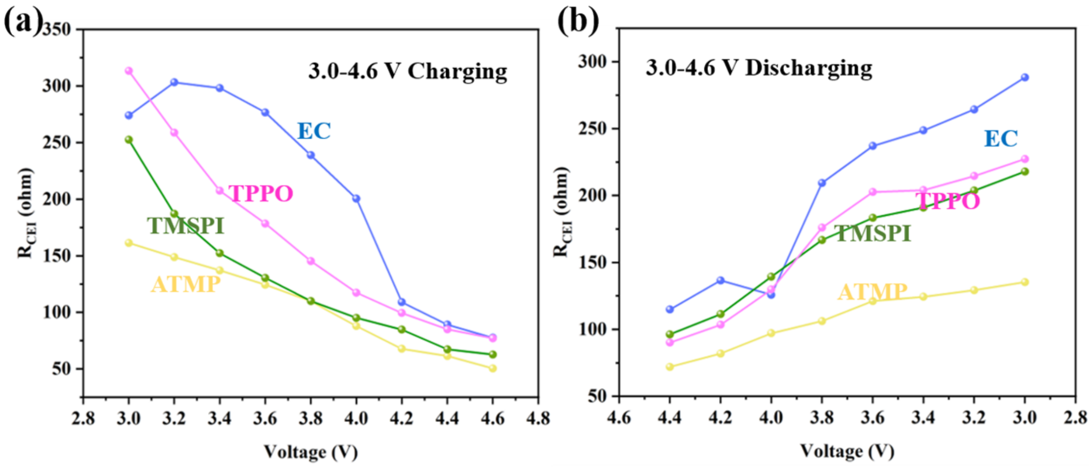

Figure S2. (a) The variation trend of R_S value during charging process from 3.0 V to 4.6 V for LED, LAED, LTMED, and LTPED. (b) The variation trend of R_S value during discharging process from 4.6 V to 3.0 V for LED, LAED, LTMED, and LTPED.

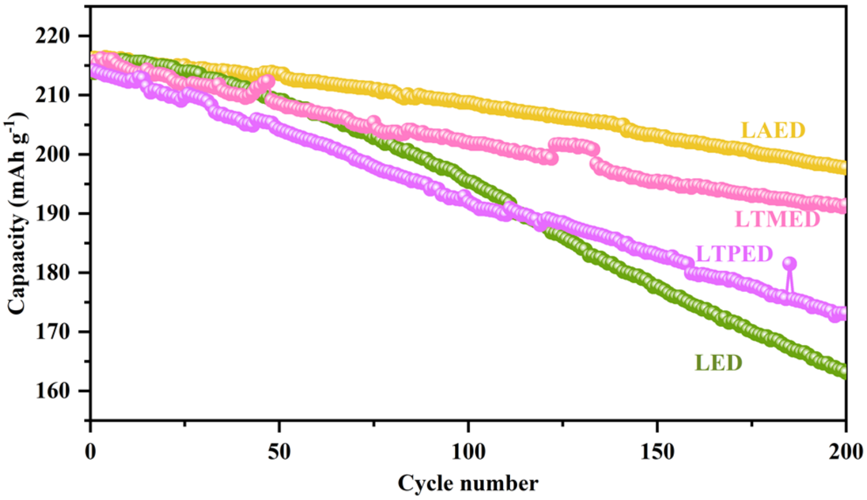

Figure S3. Cycling performance of the half-cells cycled at LED, LAED, LTMED, and LTPED electrolyte with a voltage range of 3.0-4.6 V after 200 cycles.

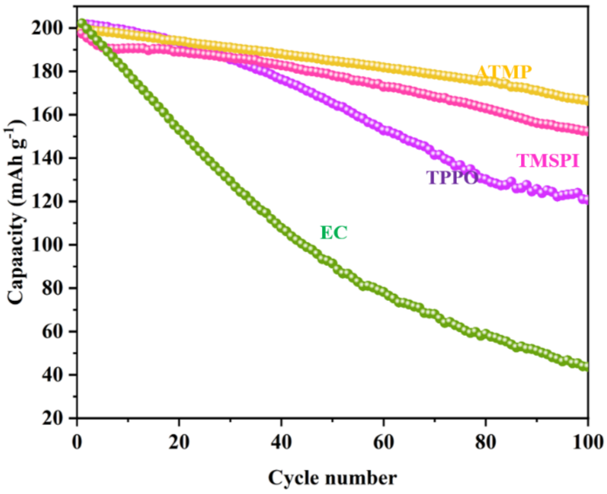

Figure S4. Cycling performance of the full-cells cycled at LED, LAED, LTMED, and LTPED electrolyte with a voltage range of 3.0-4.5 V after 100 cycles.

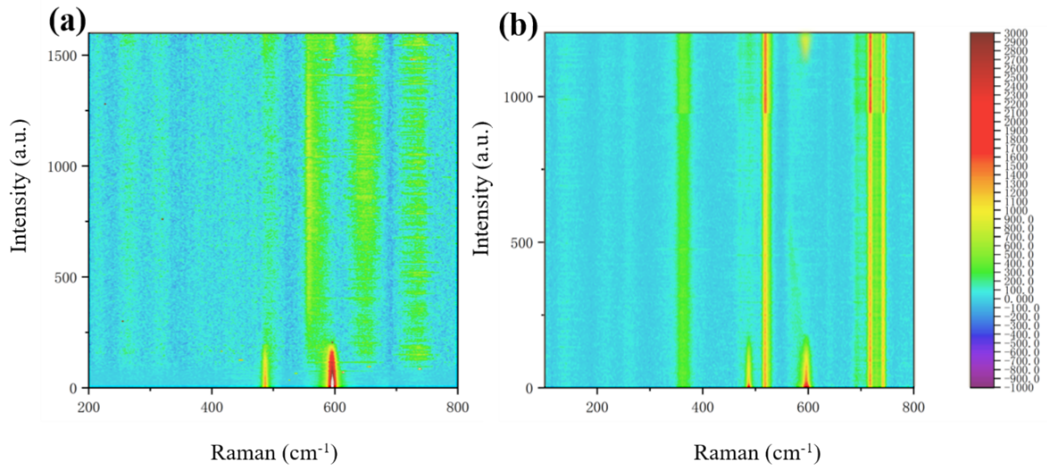

Figure S5. (a) In-situ Raman original data for the cell contains LED electrolyte. (b) In-situ Raman original data for the cell contains LAED electrolyte under 3.0-4.6 V.

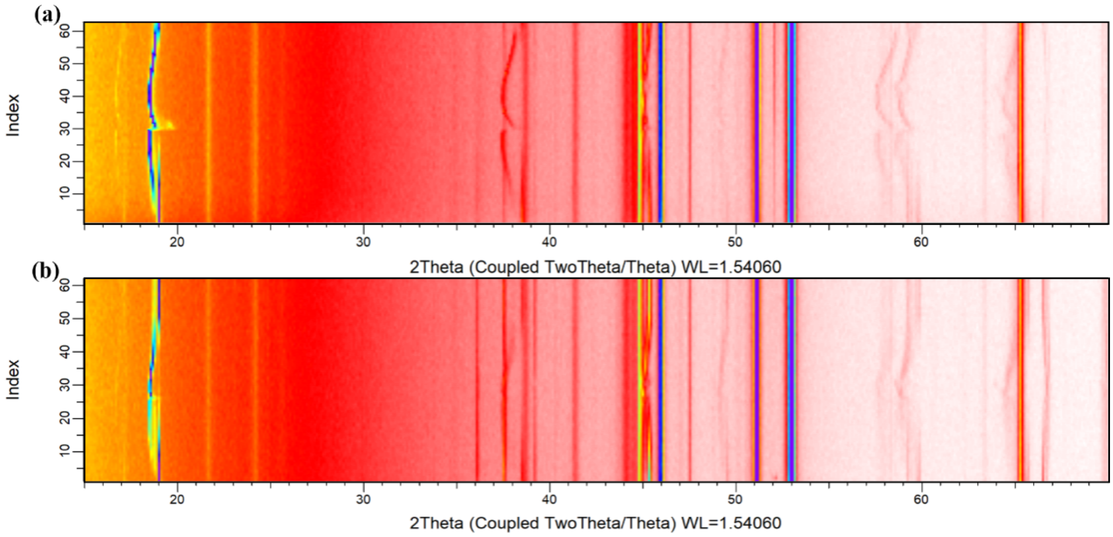

Figure S6. (a) In-situ XRD contour images for the cell contains LED electrolyte. (b) In-situ XRD contour images for the cell contains LAED electrolyte with a voltage range of 3.0-4.6 V at 0.5 C.

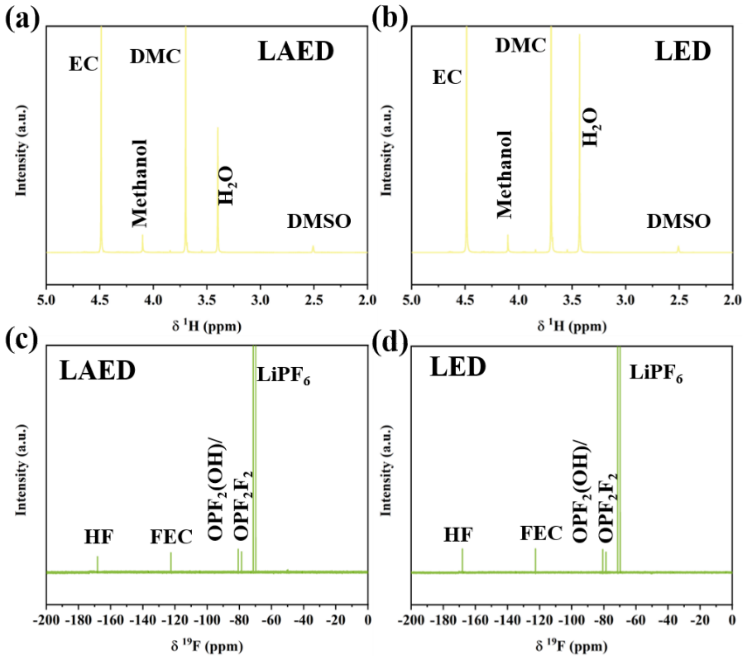

Figure S7. (a-b) ^{1}H NMR signal of anode extracted from the cell with (a) LAED and (b) LED to DMSO-d_6 solvent. (c-d) ^{19}F NMR signal of anode extracted from the cell with (c) LAED and (d) LED to DMSO-d_6 solvent.

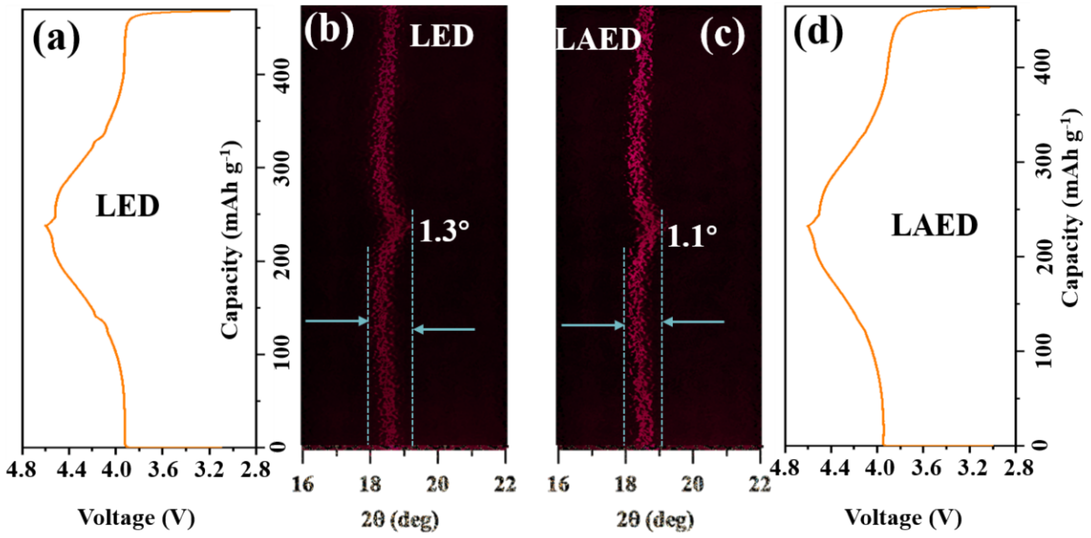

Figure S8. (a-b) Corresponding charge–discharge curves with LED electrolyte, and in-situ XRD data of 003 peak. (c-d) Corresponding charge–discharge curves with LAED electrolyte, and in-situ XRD data of 003 peak.

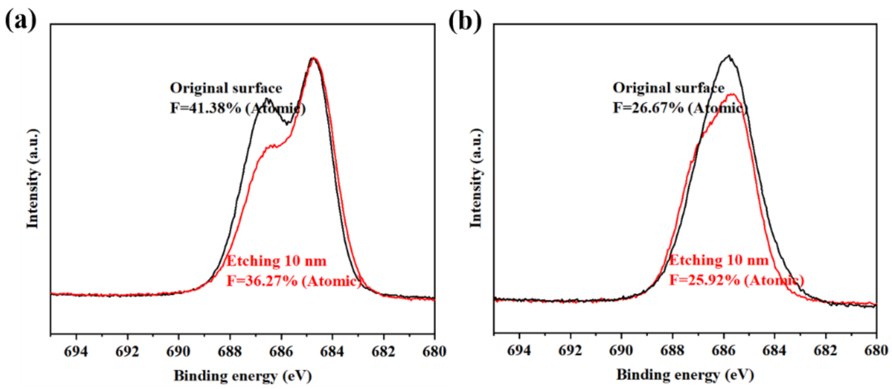

Figure S9. (a-b) XPS depth of F 1s for LCO with (a) LED, (b) LAED.

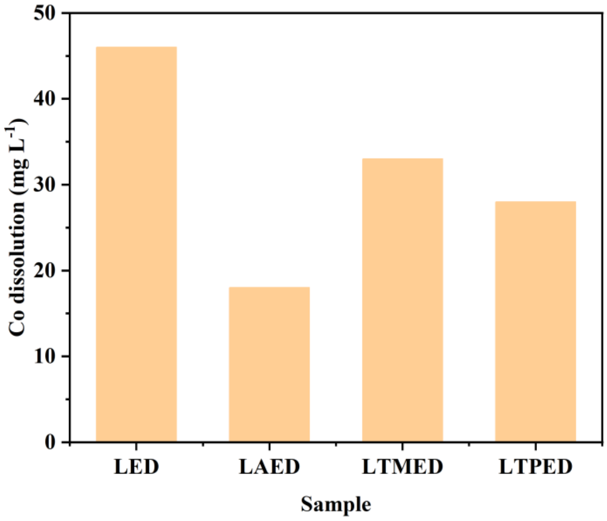

Figure S10. ICP measurements of the dissolved concentrations of Co ions in LCO with LED, LAED, LATMED, LATPED.

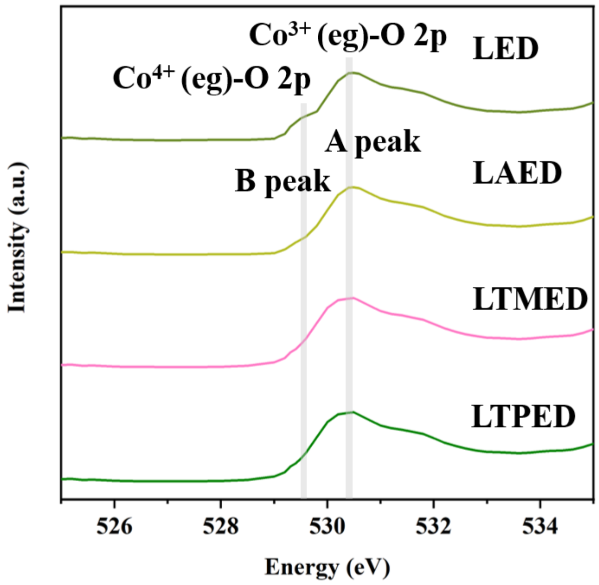

Figure S11. O K-edge XAS spectra of LCO after 50 cycles with different electrolyte.

[1] a) C. Sun, B. Zhao, J. Mao, K. H. Dai, Z. y. Wang, L. b. Tang, H. z. Chen, X. h. Zhang, J. c. Zheng, *Advanced Functional Materials* **2023**, 33, 2300589; b) C. Sun, B. Zhao, R.-d. Cui, J. Mao, K.-H. Dai, H.-Z. Chen, X.-h. Zhang, J.-c. Zheng, *ACS Applied Materials & Interfaces* **2023**, 15, 21982.
